# Practical Notes on Diagnosis and Treatment

**Published:** 1910-03-26

**Authors:** 


### Practical Notes on Diagnosis and Treatment.

**Corhea.**

Cold affusion, especially over the spine, is very beneficial, and excellent results have been obtained from the shower-bath.

**Syphilis in the Internal Ear.**

In the tertiary stage the patient may become suddenly deaf within a few days after the onset; frequently this is accompanied by vertigo, tinnitus, and attacks of vomiting, and is due to inflammation of the labyrinth.

**Visceral Pain.**

Pain caused by pressure over an organ is due to the stimulation of structures over it and not to the sensitiveness of the organ itself. Certain hyperæsthetic areas are constantly related to gastric ulcers according to the position of the latter. The stomach may move considerably, but the painful spot still remains constant in position.—*Dr. J. Mackenzie.*

**Chloretone in Chorea.**

Chloretone appears to be of great value in the treatment of chorea, attacks are shortened, and the tendency to second and third attacks seems also to be reduced. The dose ranges from five to twenty grains, but a maximum of ten grains three times a day is usually sufficient; it is given with drachm doses of petroleum emulsion. As soon as any improvement is visible—generally in two or three days—the dose should be reduced by one grain a day till only one-grain doses are given. An important adjunct to any form of treatment of chorea is the complete surrounding of the bed by screens, if in a ward, or seclusion of the patient if in a private house.—*Dr. Montague Murray.*

**Lead Poisoning.**

Beyond the usual symptoms of plumbism there may be found a tenderness over the vagus nerve in the neck, the absence of potassium sulpho-cyanide in the saliva, and a basophilic granular degeneration in the red-blood corpuscles.

**Whooping Cough.**

In the earliest stages it is common to get a high lymphocytosis in the blood of children. This might be made use of under suitable circumstances as a valuable diagnostic aid, since the lymphocytosis may be obtained before any symptoms of infection are obvious, and yet the child may be capable of spreading the disease.

**Furunculosis of the External Meatus.**

This may be due to staphylococcic infection of an abrasion, or may occur after the extensive use of bromides, and in the course of diabetes or otitis media. The pain may be relieved by the instillation of these drops: carbolic acid 6 grains, morphine hydrochlor 3 grains, glycerine one drachm. They should be used as warm as can be borne.—*Mr. E. B. Waggett.*

**Water Drinking.**

The custom of drinking a glassful of water on rising and on retiring is one to be encouraged. Many people fall short in the amount of fluid they take during the day, and this is especially true of women. The morning glass of water does not impair the appetite for breakfast as does the early cup of tea and biscuit; it also acts as a natural laxative, and tends, by cleansing the mucosa, to prepare the stomach for the meal.

